# Investigator Experiences Using Mobile Technologies in Clinical Research: Qualitative Descriptive Study

**DOI:** 10.2196/19242

**Published:** 2021-02-12

**Authors:** Kevin Christopher McKenna, Cindy Geoghegan, Teresa Swezey, Brian Perry, William A Wood, Virginia Nido, Steve L Morin, Brigid K Grabert, Zachary P Hallinan, Amy L Corneli

**Affiliations:** 1 Department of Population Health Sciences Duke University Durham, NC United States; 2 Clinical Trials Transformation Initiative Durham, NC United States; 3 Patient and Partners LLC Madison, CT United States; 4 Department of Medicine Division of Hematology University of North Carolina at Chapel Hill Chapel Hill, NC United States; 5 Lineberger Comprehensive Cancer Center School of Medicine University of North Carolina at Chapel Hill Chapel Hill, NC United States; 6 Genentech, Inc. San Francisco, CA United States; 7 Office of Health and Constituent Affairs US Public Health Service US Food and Drug Administration Silver Spring, MD United States; 8 Duke Clinical Research Institute Duke University Durham, NC United States

**Keywords:** mHealth, mobile technology, mobile clinical trials, digital health, clinical research, mobile devices, digital health technology, mobile applications, clinical trial

## Abstract

**Background:**

The successful adoption of mobile technology for use in clinical trials relies on positive reception from key stakeholders, including clinical investigators; however, little information is known about the perspectives of investigators using mobile technologies in clinical trials.

**Objective:**

The aim of this study was to seek investigators’ insights on the advantages and challenges of mobile clinical trials (MCTs); site-level budgetary, training, and other support needs necessary to adequately prepare for and implement MCTs; and the advantages and disadvantages for trial participants using mobile technologies in clinical trials.

**Methods:**

Using a qualitative descriptive study design, we conducted in-depth interviews with investigators involved in the conduct of MCTs. Data were analyzed using applied thematic analysis.

**Results:**

We interviewed 12 investigators who represented a wide variety of clinical specialties and reported using a wide range of mobile technologies. Investigators most commonly cited 3 advantages of MCTs over traditional clinical trials: more streamlined study operations, remote data capture, and improvement in the quality of studies and data collected. Investigators also reported that MCTs can be designed around the convenience of trial participants, and individuals may be more willing to participate in MCTs because they can take part from their homes. In addition, investigators recognized that MCTs can also involve additional burden for participants and described that operational challenges, technology adoption barriers, uncertainties about data quality, and time burden made MCTs more challenging than traditional clinical trials. Investigators stressed that additional training and dedicated staff effort may be needed to select a particular technology for use in a trial, helping trial participants learn and use the technology, and for staff troubleshooting the technology. Investigators also expressed that sharing data collected in real time with investigators and trial participants is an important aspect of MCTs that warrants consideration and potentially additional training and education.

**Conclusions:**

Investigator perspectives can inform the use of mobile technologies in future clinical trials by proactively identifying and addressing potential challenges.

## Introduction

### Digital Health Technologies in Health Care

Health care systems are increasingly using digital health technologies, such as smartphones, tablets, notebook computers, watches, other wearables, and mobile device apps for collecting health data and delivering health care–related services. The use of such technologies, also referred to as mobile technologies, is associated with improved outcomes for many patients [1-6]. For example, smartphone apps have facilitated diet tracking and led to significantly greater weight loss [7], and a game-based module has improved drug adherence, resulting in lower rates of side effects from chemotherapy among patients with breast cancer [8]. As health care systems continue to adopt digital health technologies in the provision of patient care, there is promise of improvement in the delivery of health services to patients in the future.

### Digital Health Technologies in Clinical Research

Digital health technologies can also be incorporated into clinical research to potentially improve efficiency, data quality, and data collection. A clear sign of the oncoming shift toward this type of technology comes from the US FDA (Food and Drug Administration), which revised its guidance on software and mobile health (mHealth) technology to encourage innovation in the area of digital health technologies [9]. Although the promise of this technology is garnering enthusiasm from investigators studying issues such as rare diseases [10], high blood pressure [11], and medication adherence [12], information is sparse on how site investigators feel about the potential value and challenges of embedding digital health technologies within clinical trials. Most of the current literature focuses on the acceptability of mHealth apps and the preferences of clinicians and patients for certain features of mobile technologies for specific types of patients [13-15]; for example, the assessment of preferences for an mHealth app to support patients with chronic arthritis [16].

### Study Objectives

Recognizing a gap in the evidence base regarding site investigator preferences and barriers, the Clinical Trials Transformation Initiative (CTTI) assessed patient and site investigator perspectives on the use of digital health technologies in clinical research (referred to as mobile technologies at the time of study implementation). CTTI previously published survey findings on trial participant preferences on mobile technologies [17]. Here, we describe site investigator preferences on such technologies. We focused on the following: (1) site investigators’ insights on the advantages and challenges of mobile clinical trials (MCTs); (2) site-level budgetary, training, and other support needs necessary to adequately prepare for and implement MCTs; (3) site investigators’ insights regarding the advantages and challenges for participants; and (4) suggestions for addressing challenges. We hope that these data will inform the use of digital health technologies in clinical trials for future investigators, particularly to inform investigators’ expectations and planning efforts and clinical research sponsors’ understanding of the challenges investigators face at the site level.

## Methods

### CTTI

CTTI is a public-private partnership cofounded by Duke University and the US FDA that seeks to develop and drive adoption of practices to increase the quality and efficiency of clinical trials. CTTI has led several projects on the use of digital health technologies in clinical research [18].

### Design and Methods

We conducted a qualitative, descriptive study [19,20] using in-depth interviews with investigators involved in the conduct of MCTs from June 8 to October 11, 2017. Interviews were conducted over the telephone by trained interviewers and were digitally audio recorded with the investigator’s permission.

### Investigator Selection and Recruitment

We purposefully sampled investigators [21] for participation in this study. They must have had experience in conducting clinical research in the United States (eg, pilot studies, observational studies, phase I-III studies, postmarketing studies, and feasibility studies) that used mobile technology. We did not focus on sampling specific types of mobile technologies; however, investigators must have used the technology to collect data versus for study procedures (eg, for recruitment, retention, or informed consent) or to collect patient-reported data only (eg, accessing internet-based surveys).

Multiple strategies were used to purposefully recruit investigators. First, we contacted sponsors and investigators of MCTs who had previously participated in CTTI interviews on the use of mobile technologies in clinical trials (and who had given permission to be recontacted for CTTI research and projects) and asked if they would be willing to identify site investigators (whom we would then invite for interview participation) or to pass along information about our study to appropriate site investigators. Second, we searched the National Institutes of Health database of privately and publicly funded clinical studies and identified investigators of trials that used mobile technologies. Third, we reviewed articles identified by a CTTI-sponsored systematic review of studies that used mobile technologies to measure clinical endpoints [22] to identify investigators. Finally, the CTTI Mobile Clinical Trials Engaging Patients and Sites project team identified investigators from within their professional networks. Study staff reached out directly to these investigators to screen them for eligibility and, if eligible, to invite them to participate in an interview.

### Data Collection

Demographic information was collected at the beginning of the interview. We explored a range of topics during the interview, including (1) investigators’ perceptions of the advantages and disadvantages of MCTs compared with traditional clinical trials for both trial investigators and trial participants and the impact of these advantages and disadvantages on clinical trial activities; (2) how to overcome any disadvantages of using mobile technologies in clinical trials; (3) site support and implementation needs, including budgetary requirements and relevant training for both study staff and trial participants; (4) experiences with investigator and trial participant access to study data; (5) additional Institutional Review Board (IRB) requirements and concerns when using mobile technologies in clinical trials; and (6) guidance for other investigators interested in conducting MCTs. We did not explore perspectives on MCT study designs, the use of mobile technologies for potential participant recruitment or consent, or the incorporation of mobile technologies to enhance patient-reported outcomes.

### Data Analysis

We used descriptive statistics to summarize the demographic data and applied thematic analysis [23] to analyze investigators’ narratives. All interviews were first transcribed verbatim following a standardized transcription protocol [24]. Investigators’ narratives were analyzed using a 2-stage deductive and inductive analysis approach. First, 2 analysts applied deductive structural codes (based on interview topics and organized by research objectives), such as *advantages of MCTs* and *disadvantages of MCTs*, using NVivo 11 [25]. Intercoder agreement was assessed for 25% (3/12) of the transcripts. Any discrepancies in code application were resolved through group discussion; edits were subsequently made to the codebook so that it could be used in the coding of future transcripts, and previously coded transcripts were recoded based on the modified codebook. Second, after the initial deductive coding was complete, coding reports were generated and reviewed by analysts to identify emergent themes; these themes were subsequently coded using NVivo 11 using content codes such as *greater study-related burden on participants* and *challenges of real-time data access for participants*. Summary reports of the content codes were generated and reviewed by analysts. After discussions with analysts, potential themes and the nuances of each theme were examined, and final themes and subthemes were identified based on their salience. Analysts wrote analytical summary reports to describe all themes and subthemes, together with illustrative quotes.

### Ethics

The Duke University Health System IRB determined that the research met the requirements for exemption from further IRB review. All investigators received an informational sheet before study participation that explained the study in detail, including its purpose, risks, and benefits.

## Results

### Study Population

We interviewed 12 site investigators who were diverse in clinical specialties, affiliations, types of clinical trials conducted, and years of experience in conducting both traditional and MCTs. Half of the investigators (6/12, 50%) had experience with observational studies using mobile technologies, whereas more than half (8/12, 67%) had also conducted Phase III trials using mobile technologies (both registrational and nonregistrational). A high percentage of investigators had experience in conducting device acceptability and/or feasibility studies (9/12, 75%) and device validation studies (8/12, 67%; Table 1). In addition, 6 investigators had conducted both industry-funded and investigator-initiated clinical research, 5 had conducted only industry-funded research, and 1 had conducted only investigator-initiated research.

**Table 1:** Investigator demographics (N=12).

Demographics		Values, n (%)
**Clinical specialty**		
	Cardiology	2 (15)
	Hematology	2 (15)
	Psychiatry	2 (15)
	Endocrinology	1 (8)
	Family medicine	1 (8)
	Internal medicine	1 (8)
	Internal medicine and gastroenterology	1 (8)
	Immunology	1 (8)
	Neurology	1 (8)
	Oncology	1 (8)
	Pharmacy	1 (8)
**Affiliation**		
	Academic institution	7 (58)
	Dedicated research site	2 (17)
	Other^a^	3 (25)
**Years of experience with traditional clinical research**		
	1-10	5 (42)
	11-20	3 (25)
	21-30	4 (33)
**Types of traditional clinical research^b^**		
	Phase I	7 (58)
	Phase IIa or IIb	9 (75)
	Phase III (nonregistrational)	8 (67)
	Phase III (registrational)	8 (67)
	Observational studies	9 (75)
	Other^c^	5 (42)
**Years of experience with mobile clinical research**		
	1-5	6 (50)
	6-10	5 (42)
	>10	1 (8)
**Types of mobile clinical research**		
	Phase I	4 (33)
	Phase IIa or IIb	3 (25)
	Phase III (registrational)	6 (50)
	Phase III (not registrational)	6 (50)
	Observational studies	6 (50)
	Device feasibility or acceptability studies	9 (75)
	Device validation studies	8 (67)

^a^Investigators reported affiliations with a clinical trial start-up, clinical practice and research entity, and a community-based large multispecialty clinic.

^b^Investigators reported all that applied.

^c^Investigators reported experience with Phase IV trials, embedded qualitative research, telemedicine, patient registries, and interventional trials in addition to at least one other type of research listed.

Investigators had experience using a wide range of mobile technologies in clinical research. The most frequently reported technologies were continuous glucose monitors (3/12, 25%) and activity or sleep monitors (3/12, 25%). A variety of endpoints were also described. The most frequently reported endpoints were medication compliance (3/12, 25%) and blood sugar levels (3/12, 25%; Table 2).

**Table 2:** Investigators’ use of technology: type, endpoint, and therapeutic area of investigation (N=12).

Use of technology			Values, n (%)
**Type^a^**			
	Mobile app^b^	7 (58)	
	Commercial grade activity and sleep monitor	3 (25)	
	Continuous glucose monitor	3 (25)	
	ePRO^c^ device^d^	3 (25)	
	Electronic pill bottle	2 (17)	
	Ingestible sensor with patch	2 (17)	
	Ambulatory blood pressure monitor	1 (8)	
	Holter monitor	1 (8)	
	Implantable cardioverter defibrillator	1 (8)	
	Mobile spirometer	1 (8)	
	Tablet-based video monitor	1 (8)	
	Wearable EKG^e^ patch	1 (8)	
	Wireless weight scale	1 (8)	
**Endpoint^f^**			
	Blood sugar	3 (25)	
	Medication compliance	3 (25)	
	Blood pressure	2 (17)	
	Change in heart rate	2 (17)	
	Heart rhythm	2 (17)	
	PRO^g^	2 (17)	
	Steps or activity level	2 (17)	
	Change in aerobic capacity	1 (8)	
	Pulmonary artery pressure	1 (8)	
	Ratio of insulin and glucagon levels	1 (8)	
	Sleep quality	1 (8)	
	Spirometry	1 (8)	
	Visual acuity	1 (8)	
	Weight	1 (8)	
**Therapeutic area**			
	Cardiology	4 (33)	
	Neurology	4 (33)	
	Diabetes	3 (25)	
	Hemophilia	1 (8)	
	Impact of energy drinks (measure of activity and sleep)	1 (8)	
	Ophthalmology	1 (8)	
	Oncology (undergoing chemotherapy)	1 (8)	
	Pulmonology	1 (8)	

^a^Investigators reported all types of mobile technology they used in any of their clinical research studies.

^b^Investigators reported using mobile apps either as a data collection tool or as a hub to receive and send data from a linked mobile device in addition to at least one other listed technology (not including ePRO).

^c^ePRO: electronic patient-reported outcome.

^d^Investigators reported using patient-reported outcome data in addition to at least one other listed technology (not including mobile apps).

^e^EKG: electrocardiogram.

^f^Investigators reported all study endpoints measured using a mobile technology.

^g^PRO: patient-reported outcome.

### Site Investigator Insights on the Advantages and Challenges of MCTs

#### General Advantages of MCTs Compared With Traditional Trials

Investigators most commonly cited 3 advantages of MCTs compared with traditional clinical trials: (1) more streamlined study operations, including data collection; (2) remote data capture; and (3) improvement in quality of studies and data collected (Table 3).

**Table 3:** General advantages of mobile clinical trials.

Advantages	Participant examples and descriptions	Participant quotes
More streamlined, simpler study operations and data collection	More efficient to do continuous follow-up remotely than episodic follow-up in clinic Less costly to conduct (fewer clinic visits and fewer research coordinators) Easier to manage more trial participants	“Typically with traditional clinical trials there is a schedule of in-clinic follow-up, and there are windows within which patients fall in or out of if you get them back at the right time or not. And, it’s just a lot more efficient when you can do a lot of this follow-up on a remote basis because it ensures, in many ways, a more continuous follow-up rather than this episodic follow-up. And, makes it easier to get key endpoints assessed within a follow-up window, without the reliance on having patients travel sometimes great distances to get in for a clinic follow-up.”
Remote data capture	Easier to collect a higher volume of data because not restricted to data collection snapshots during clinic visits Continuous or higher frequency data collection provide more accurate account of trial participants’ experiences Remote monitoring decreases burden on trial participants Provides greater insights into trial participants’ experiences because of real-world data Can include more endpoints because of higher volume of data	“Mobile devices can make it easier. They can serve as a really good, valid substitute of what could otherwise be a very cumbersome process. For instance, doing an ambulatory blood pressure monitor, where it’s being checked like every 15 minutes for a 24-hour period. It’s a little easier than having a patient put their arm in a cuff manually every 15 minutes, right, and checking it. So there are times when your only valid option, the only rational option, is to use a mobile device.”
Improvement in quality of studies and data collected	Designed around trial participants rather than researchers or research sites, which increases the likelihood of generating more applicable real-world study results Potential to provide better, more objective, higher frequency, and possibly more sensitive assessments of trial participants than traditional trials Enhanced data quality because of higher frequency data points, less reliance on activity logs Enhanced ability to deliver quality data—particularly compliance data—to sponsors, which may make it more likely that sponsors will come back to the research site with additional studies Minimizes geography barrier, which can enhance diversity of participant sample	“It’s easier to make decisions because I've got better quality data. The statistical analysis on the trial should be superior because we've got higher-level data. We’re having to throw out fewer data points because of subjective reasons where you're looking at a piece of data and saying, ‘That can't possibly be accurate,’ but you've got an increased accuracy with the mobile data collection. So there are things like that that make running the research trial easier because of the mobile data collection. But it's more in being able to trust the data and in your analysis of the data.”

#### Investigator-Specific Advantages of MCTs Compared With Traditional Trials

All 12 investigators spoke about their willingness to lead another MCT, with many stating that they believe MCTs are the future of clinical trials. Almost all of these investigators tied their willingness to participate in future MCTs to the perceived benefits of these trials compared with traditional clinical trials, particularly the investigator’s role in responding to remote data capture and improvements in the efficiencies of study procedures (Table 4).

**Table 4:** Investigator-specific advantages of mobile clinical trials.

Advantages	Participant examples and descriptions	Participant quotes
Remote data capture	Ability to intervene (ie, communicate with) with trial participants as needed based on real-time data Enhanced monitoring of trial participants’ compliance to study procedures Better systems for notifications and reminders to enhance compliance Easier to conduct assessments and make protocol-based management decisions remotely	“It’s of tremendous importance because we are dealing in real-time versus a month later or six months later. And if you see something that’s not right you can intervene immediately and take care of it.” “We can do small things to help patients perform better in the study. So we can do things like we use push notifications and reminders as well and so that patients, if they don’t do an activity for a day, then they can get a push notification on their study device saying, ‘Don’t forget to track your symptoms today’... or if they forget to wear a watch overnight to track their sleep, the next morning, we can remind them to wear their watch the next day.”
Improvement in efficiency of study procedures	Ability to more objectively monitor compliance with study procedures and medication adherence Real-time access to data Better management of trial participants because of continuous access to data	“It’s really essential because it’s what [an investigator] is basing [a] treatment decision upon – having access to the information to do it.… so that they could implement the protocol driven medication changes based on change in pulmonary pressure.”

#### Investigator-Specific Challenges of MCT Compared With Traditional Trials

Investigators reported that operational challenges, time burden, technology adoption barriers, and uncertainties about data quality made MCTs more challenging than a traditional clinical trial (Table 5).

**Table 5:** Investigator-specific challenges with mobile clinical trials.

Challenges	Participant examples and descriptions	Participant quotes
Operational challenges and time burden	Additional time required to review higher volumes of data in real time Additional study procedures necessary such as following up on missing data Increased time needed for setting up technologies and linking devices to specific users Large amount of time needed by site staff in maintaining technologies, such as charging and storing devices and troubleshooting problems with malfunctioning devices Large amounts of data to be managed Additional time needed for training on the use of various participant databases Time often not reimbursed	“Usually the beginning of the study we’ll just get a protocol synopsis, so we won’t get all of the information. And oftentimes we’ll make a decision about whether we take a study on the basis of a protocol synopsis... It might sound like we’re going to be collecting this information. And then when you find out how they’re going to be collecting it, what type of device they’re using, what the burden is on the staff, there can be moans and grunts at an investigator meeting.”
Technology adoption barriers by both staff and trial participants	Difficulties exist among trial participants in using some technologies (eg, feel and comfort of the device, complexity of the participant interface), which can impact compliance Time needed to train staff and participants on how to use and troubleshoot the technology	“[Devices] are complicated to use. They are not terribly user friendly... Research trials are harder and now you end up with a patient in a research trial and the device causes that patient to have to drop out of that trial.”
Uncertainty about data quality	Potential for new data biases exists when participants have access to their personal data The real-world nature of trial is reduced when more study staff must intervene with trial participants to address data quality issues The clinical meaningfulness of the data is questioned, including how much data should be collected to inform the outcome Data variation making the analysis plans more complicated Technical issues may compromise data quality (eg, wireless connectivity in rural areas)	“[Not allowing participants access to their data is] a plus and a minus. I think you worry that if they see their own data, if it affects what they're doing and bias the studies. You might have people all of a sudden trying to get to a certain number of steps a day or behaving differently because they're monitoring their steps, and so [researchers] avoided that by not having the patients see their data. I think that created some obstacles because then you don't have any real-time confirmation that the data is being transmitted and also the patient doesn't feel like they're getting any benefit out of wearing it.” “Because of a device malfunction, that patient's data has to be thrown out, and that's just terribly unfortunate when that kind of a thing happens.”

#### Suggestions for Overcoming the Challenges With MCTs

All 12 investigators interviewed had concerns about added site burden in MCTs and made recommendations or observations about ways to minimize these concerns. The most common suggestion for sponsors was to establish and foster collaborative relationships with sites and investigators. One frequently recommended strategy for this was sponsor solicitation of input from sites and investigators about vendors and technology selection. A few investigators also recommended that sponsors provide complete information about the type of device and how technology will be used before any contractual agreements.

Many investigators mentioned the importance of being able to properly address challenges presented by the chosen technology. Investigators commented that appropriate device selection could help prevent challenges, that sponsors should compensate sites appropriately for added staff time involved in device training and troubleshooting, and that sponsor provision of an adequate number of surplus devices would help prevent issues with data loss in the face of device malfunction. In addition, a few investigators highlighted the importance of ensuring that investigators have a thorough understanding of the technology, with one specifying:

My recommendation to other investigators would be know your device, know all the nuances of the device...

Many investigators have emphasized the importance of having systems in place to ensure data integrity when using mobile technologies. One investigator described monitoring data for outliers as a form of quality control on collected data:

We do have much more data coming in, so it is easier to sort of identify outliers. Right? So if we’re using a wireless scale and every day, we get a measurement of 200 pounds plus or minus a couple of pounds and all of a sudden, we get a measurement of 80 pounds, you can sort of identify some of those outliers as well.

### Site-Level Budgetary, Training, and Other Support Needs

#### General Needs of MCTs

Technical support was the most frequently mentioned type of assistance needed when implementing a trial using mobile technologies. One investigator also spoke about the utility of technical support when technologies are used in trials in ways that were not initially intended by the manufacturers:

We need a lot of support, more so than usual... And typically, we're not using the devices exactly as intended, so for this ECG [electrocardiogram] patch, it's designed to put on in the hospital or in a clinic setting. And we were having the participants do it alone. You have to work with the manufacturers about the educational material, making sure technically it's going to work that way, and what's important and not. And I'd say with all of the manufacturers we've had to work with, it's required a real partnership, not just a vendor kind of, “We’re just going to purchase this and use it” type of relationship.

Budgetary support was also frequently mentioned. Several investigators indicated that they were not adequately prepared to plan for a trial involving mobile technologies from a budgetary perspective, specifically as it related to allotting appropriate funds for staff time required.

#### Suggestions for Technology Support From Sponsors

Many investigators have suggested that sponsors should provide technical support for activities such as initial setting up of technologies, monitoring of trial participants, data management, and ongoing troubleshooting of technologies. Several also described the need for sponsors to assist with technology-related issues, such as having a call center, or by providing a third-party vendor hired to troubleshoot device-related issues.

Device training was also mentioned by investigators as a provision that should be organized by the sponsor, as was device procurement. One investigator described the sponsor support he received with his MCTs:

The sponsors provided training on aspects of device functioning, and how to interrogate the device, and how to access the data through their usually secure web-based portals and so on and so forth. So they provided training on the system. And again, they generally have people in the field who provide troubleshooting; so if there is a problem with not being able to connect to the systems or some other issue, they usually have people in the field that can support the staff in terms of troubleshooting.

A few investigators suggested that sponsors should conduct a technology assessment to ensure that the best technology is selected or provide more specific information about technologies to be used in the trial when available, such as any potential and past malfunctions and issues associated with devices. In addition, one investigator suggested that sponsors partner with more advanced commercial developers who could better ensure that trial participants would be optimally engaged when interfacing with devices.

A few investigators emphasized fit appropriateness for specific trial participant populations. For example, investigators described selecting technology that is most appropriate for older trial participants who might have less fine motor dexterity in the hands and offering support that was sensitive to the specific needs of certain populations. Another investigator mentioned that sponsors should provide more devices to sites that are historically adept at enrolling trial participants so that backup devices could be issued in the event of a device malfunction without causing any delay in data capture. An investigator said:

Things that we could do to have an immediate impact on the conduct of these trials, one, provide more of the devices to well-enrolling sites. If I have more devices and I have a patient with a tech support issue right now, I can swap out the device instantaneously and now take my time at getting this other device analyzed for tech support, but if that's the only device, then it's everyone's brow is sweating. “Are we losing data right now?” “How quickly can we get this fixed?” “The patient's getting upset.” “I need to go pick up my kids.” So having more devices would absolutely help.

Finally, investigators stressed the importance of sponsors ensuring that technology support was easily accessible throughout the life of a trial, staffed by actual people, and centralized so that each site would not be individually responsible for troubleshooting when trial participants have challenges with technologies.

#### Suggestions for Budgeting

Many investigators recommended that researchers develop a clear and comprehensive budget that contains adequate compensation for the purchase and storage of devices and staff time spent in training and troubleshooting issues that may arise with technologies. Most of these investigators acknowledged challenges with budget planning, as unforeseen expenses were often mentioned as a common feature of clinical trials that involved mobile technologies. Many investigators recommended budgeting for staff time related to various mobile-specific trial activities, as this was the biggest additional expense when compared with traditional trials. They also recommended budgeting for costs directly related to procuring and managing technologies, including purchasing devices, device repair and storage, device rental, and setup fees associated with certain technologies. A few investigators specifically mentioned flexibility on behalf of sponsors as key to accommodating these types of trials, given how relatively new they are to clinical research. One investigator stated:

I do tend to underbudget because it's hard to take into account all of the eventualities that might occur, and the other thing is, it's hard to justify that. If you're trying to write a budget and you say we have this many hours of this many people, and so forth, and then you can say, in your own head, you can say I'm going to add 50 percent because it never goes as planned, and then it turns out you really should have doubled it. I think sponsors need to understand that... I know they're all focused on trying to get the biggest bang for their buck, but you don't want to underfund the study so that you leave it hobbled and struggling to meet its aims.

#### Additional Suggestions

##### Training

Almost all investigators spoke in depth about how to improve training for staff and trial participants. Some investigators suggested that sponsors should allocate more time to training to ensure that site staff are familiar and comfortable with the technologies that trial participants will use. Many mentioned the ideal role that sponsors play in the provision of comprehensive training on devices. One investigator described the various training sponsors had provided in previous trials, including how to share data collected from the device.

The study sponsors provided training on aspects of device functioning and how to integrate the device and how to access the data through their secure web-based portals. Investigators also recommended ways in which future site training on MCTs could be enhanced. Several investigators thought it would be beneficial for training to include more hands-on time with technologies and in-person teaching (vs web-based training). Some also recommended that training should provide device-specific materials that could be referenced throughout the life of the study, with ad hoc access to education and feedback mechanisms related to technology functionality. To enhance the efficiency of device training, some felt that web-based training should be self-paced and optional (vs required) and also indicated that training should be optional for technologies that sites were already familiar with. Investigators felt that training should cover strategies to address device malfunction, study monitoring of real-time data, and suggest ways to improve staff empathy for trial participants. Some also noted that training should aim to provide sponsors, investigators, and study staff with a thorough understanding of technologies before trial initiation.

##### IRB

Most investigators noted that although their IRBs did not raise any concerns regarding the use of mobile technologies in the research, several said sharing the following information with IRBs would be helpful: data security, including how trial participants will securely share data collected from their device with investigators; participant safety, including how actionable data shared by participants would be monitored by the study team; and certain types of documentation related to the technology, such as proof of investigational device exemption from the FDA (when appropriate).

### Site Investigator Insights on the Advantages and Challenges for Trial Participants

#### Advantages to Trial Participants

Many investigators felt that MCTs can be built around the convenience of trial participants, rather than the investigator, and, therefore, can decrease the burden of trial participation. Investigators also posited that individuals may be more willing to participate in MCTs because they can participate from home, rather than have visits that are clinic based, as in traditional trials. Investigators pointed out that trial participants have direct access to data collected about them, which may enhance engagement throughout the life of a trial (Table 6).

**Table 6:** Investigator perspectives on the advantages and challenges of mobile clinical trials for trial participants.

Advantages and challenges			Participant examples and descriptions		Participant quotes
**Advantages**					
	Trial participant access to data	Potential to drive participant engagement Increases participant sense of agency Improves management of disease or health condition		“Frankly, it’s the model of care that I think we should be moving to across the board because it improves patient engagement; it improves patient satisfaction; patients feel like they are regaining some control over their life or their disease.”	
	Decreases burden on trial participants	Reduces in-person screening Fewer visits necessitated with remote monitoring		“It becomes much less of a burden to them…instead of them having to come in at times where they otherwise wouldn't for routine care, or a follow up…all the monitoring, all the communications, everything can occur around their schedule, and at their home, where it's convenient for them.”	
	Data capture and monitoring	Automated notifications and reminders, thereby improving compliance Real-time data monitoring allowing trial participants to be reached and receive interventions Less intrusive for participants		“That automated process is going to be easier for the patient in the long run... It would be pretty easy for us to show how to do a finger stick blood sugar and just tell the patient, ‘You have to do this nine times. Before and after each meal, and then at least one random time.’ That kind of thing. Or at least nine times a day... or we could put a continuous glucose monitor in them, which would take us probably half an hour. And [this is] harder for us, but much easier for them.”	
**Challenges**					
	Additional burdens on trial participants	Intrusiveness of data capture to daily activities Technology management and upkeep needed (eg, charging devices) Education about technology use needed Additional burden could challenge recruitment and retention		“It’s one more thing they have to manage and either keep on their person or keep nearby. It can be disruptive. If they want to go out to the movies and they’re scheduled to have something, some questionnaire that’s going to be every night at 7:00 PM they might have to work their schedule around it. There has to be built in relays in these things to allow for patients to have some flexibility. And then any time you add complexity to the picture, it will make a patient less likely to want to do a study.”	
	Technology adoption barriers	Detrimental impact on participation, data quality, interest, and satisfaction because of cumbersome design and functionality of the technology Can be difficult for some participants to follow device instructions Can inhibit potential trial participants from joining the study or using the technology correctly after enrolled because of unfamiliarity with or concern about use (eg, geotracking features) Can be invasive or uncomfortable		“I think that if you have a participant who is, for whatever reason, frustrated with the device or having technical problems with the device, that person may actually become less engaged with the study or may even drop out of the study.”	
	Real-time access to data possibly impacting the behaviors of trial participants	Exposure to data may change the behavior of trial participants Reactions to inaccurate data may adversely affect the behavior of trial participants (eg, noncompliance reported when the participant had previously been compliant) Retention could be affected when data show no improvements to health Trial participants may misinterpret the meaning of the data and could misuse the information in some way		“I think that’s a bit of a disadvantage because it may hinder their mood or their willingness to seek out additional care or continue on what they’re doing if they don’t see a change in their progression, if these mobile tools are giving them feedback.”	

#### Challenges for Trial Participants

Many investigators stated that MCTs pose additional burdens on trial participants (Table 6). Challenges included placing additional burdens on participants, barriers when interfacing with technology, and the potentially negative implications of trial participants being able to access data collected about themselves. Investigators noted that these additional burdens and challenges can lead to greater participant dropout or challenges for recruitment, particularly when trial participants are not motivated to accept this added burden.

#### Suggestions for Reducing the Trial Participant Burden

Nearly all investigators suggested identifying methods to decrease trial participant burden when taking part in MCTs. Investigators recommended that the technologies selected for use in the trial should be as user friendly as possible. One investigator said:

Ideally, you make the device and the trial as simple as possible so as little as possible of the burden of understanding the device and how to make it work is on the patient... They put it on, and they wear it, and that's their role.

Several investigators suggested that all training for trial participants on the use of technologies should be participant centric. Two examples included avoiding assumptions about trial participant preferences and capabilities, and co-designing training with actual participants. Several investigators extended this concept of participant-centricity beyond training and expressed that MCT study designs overall should have a strong focus on being participant centered. For some, this meant patient involvement from the inception of the study and more access to trial developments and results.

In addition, investigators emphasized that device training for trial participants should instruct participants on not only how to use the device but also what type of information is being collected and the significance of that data to the trial being conducted.

## Discussion

### Principal Findings

We interviewed 12 investigators about the use of mobile technologies in clinical trials, and all the 12 were enthusiastic about the promise of these technologies, indicating their willingness to participate in other MCTs. Investigators described MCTs as the future of clinical research, and many advantages of using mobile technologies were identified, including benefits related to more streamlined study operations, remote data capture, and improvement in the quality of studies and collected data. However, challenges with MCTs were also described, including budgetary issues for both devices and staff time, time burdens for monitoring data and troubleshooting devices, and potential data quality issues and biases. Investigators frequently cited a need for technical support for using technologies in clinical trials and extra training and money in the budget to account for tech-related expenses. To enhance the success of MCTs, sponsors would benefit from establishing and fostering collaborative relationships with sites and investigators and soliciting input about vendors and technologies to be used.

Investigators believed that MCTs have the potential to decrease some types of burden (fewer site visits and remote monitoring) but will increase other types of burden (technology management and upkeep). Although access to real-time data could motivate and engage trial participants, concerns exist about the possibility of data misinterpretation or discouragement of use because of malfunctioning devices. Using simple, user-friendly technologies and providing training to participants is recommended.

### Limitations

Although sample sizes with qualitative research are small, the experiences and lessons learned from investigators who have used mobile technologies in clinical research can nonetheless be used to help other investigators consider the use of and prepare for MCTs. Interviews with a different group of investigators could yield different or additional benefits, challenges, and recommendations. For example, investigators using different types of technologies that are more novel and used less frequently among patients and providers might lead to additional considerations and implications for MCTs. Future research should continue to explore and document investigator and participant experiences using mobile technologies in clinical trials.

### Conclusions

The benefits of MCTs can best be realized if digital health technologies are used in a way that recognizes and addresses the day-to-day operational considerations at investigative sites. This is accomplished through stakeholder engagement, including site investigators, sponsors, and trial participants as equal partners, from the earliest stages of trial planning. Technology selection, instructing participants on how to use technology, troubleshooting of technologies by study staff, and sharing of information from technologies with investigators and participants are all important aspects of MCTs and may require additional dedicated effort, budgetary considerations, and training. The lessons identified in this paper helped inform CTTI recommendations on the use of digital health technologies in clinical research [26]. These recommendations can help both investigators and clinical research sponsors to proactively identify potential challenges and conduct high-quality MCTs.

## Abbreviations

CTTI: Clinical Trials Transformation Initiative
FDA: Food and Drug Administration
IRB: Institutional Review Board
MCT: mobile clinical trial
mHealth: mobile health
